# Low lymphocyte to high-density lipoprotein ratio predicts mortality in sepsis patients

**DOI:** 10.3389/fimmu.2023.1279291

**Published:** 2023-10-12

**Authors:** Wanjun Liu, Qian Tao, Jun Xiao, Yijun Du, Tianrong Pan, Yue Wang, Xing Zhong

**Affiliations:** ^1^ Department of Infectious Diseases, The Second Affiliated Hospital of Anhui Medical University, Hefei, Anhui, China; ^2^ Department of Endocrinology, The Second Affiliated Hospital of Anhui Medical University, Hefei, Anhui, China; ^3^ School of Clinical Medicine, Anhui Medical University, Hefei, Anhui, China

**Keywords:** lymphocyte, lipoprotein, sepsis, inflammation, immunity, prognosis, mortality

## Abstract

**Background:**

The lymphocyte-to-high-density lipoprotein (HDL) ratio (LHR) is associated with both inflammation and immunity, and may have the potential to predict the prognosis of sepsis. Our study aimed to evaluate the relationship between LHR and sepsis-related mortality.

**Methods:**

We collected data from the Medical Information Mart for Intensive Care IV (MIMIC-IV, version 2.2) database by targeting patients who met the Sepsis-3 criteria and recorded the absolute values of lymphocytes and HDL after admission. We then used restricted cubic splines based on logistic regression to simulate the relationship between the LHR and 90-day mortality. Subsequently, the hazardous threshold was derived based on the mortality curve, and further evaluations were performed using different methods and data sources for hazardous threshold.

**Results:**

We ultimately included 1027 eligible patients from the MIMIC-IV database and described the nonlinear relationship between LHR and 90-day mortality. Based on the curve, an LHR of ≤ 0.6 indicated harmful threshold, and the odds ratio for mortality was 1.74 (*P*=0.001). The outperforming hazard was particularly marked in patients with chronic lung disease and remained consistent after adjusting for baseline data and validating multiple data sources.

**Conclusions:**

The LHR has prognostic value in patients with sepsis, and an LHR ≤ 0.6 is a deleterious load that increases mortality.

## 1 Introduction

Sepsis is a syndrome of multiple organ dysfunction caused by infectious dysregulation, with complex and diverse etiologies and unpredictable clinical outcomes (1, 2). As a result of medical research, biomarkers for predicting sepsis mortality are continuously being discovered (3–5), however, lymphocytes and high-density lipoproteins (HDLs) are common laboratory indicators in clinical practice, and both have been demonstrated to be associated with sepsis mortality in previous studies (6, 7). Recently, we found that the absolute lymphocyte-to-high-density lipoprotein ratio (LHR) is a novel inflammatory marker; therefore, we hypothesized that LHR may be associated with sepsis prognosis.

Lymphocytes are associated with septic prognosis, and lymphopenia often indicates poor prognosis. Patients with sepsis exhibit adaptive responses that induce immunosuppression. In this process involving a wide and complex range of cellular changes, lymphocyte counts decrease; this involves B cells (8, 9) and all types of T cells (CD4, CD8, and Natural Killer), except T regulatory cells (10, 11), which are related to apoptosis, excessive extravasation, and aberrant recruitment to sites of inflammation, along with hampered egress to the periphery (12–14). Endothelial dysfunction and immunothrombosis play key roles in the pathological evolution of severe sepsis (15). HDLs can inhibit cytokine-induced expression of endothelial cell adhesion molecules and have anti-inflammatory, antioxidant, and antithrombotic properties (16). The LHR is a novel marker of inflammation. Several studies have found that LHR, as an inflammatory marker, can effectively predict metabolic syndrome (MetS) and is positively correlated with several metabolic risk factors (17).

To date, no study has investigated the relationship between the LHR and sepsis. Given that sepsis is a known state of systemic inflammation, we hypothesized that LHR may serve as a predictor of sepsis in patients. Therefore, the aim of this study was to investigate the association between LHR and sepsis, and to further explore the predictive value of LHR for mortality 28 days after sepsis.

## 2 Methods

### 2.1 Population and data sources

This exploratory retrospective cohort study used electronic health record data from the Medical Information Mart for Intensive Care IV (MIMIC-IV, version 2.2, data were collected between 2008 and 2019) database (18), a vast open-access database containing more than 70,000 admissions to the intensive care unit (ICU). The MIMIC is the first publicly available database for critical care settings that provides a comprehensive compilation of clinical data and well-designed physical views. Thus, the MIMIC database was initially used to investigate the distribution and patterns of LHR. However, additional evidence is required to demonstrate the generalizability of our findings. To perform external verification, we used two datasets with distinct origins. These datasets originated from two European datacenters: the University of Amsterdam Medical Center database (Amsterdam UMCdb, version 1.0.2, data were collected between 2003 and 2016) in the Netherlands (19) and the Salzburg Intensive Care database (SICdb, version 1.0.6, data were collected between 2013 and 2021) in Austria (20). Permission to use these data was granted by https://physionet.org/, and the record ID is 39691989. These datasets have received ethical approval from official institutions. As the data is de-identified, we downloaded the complete files and were authorized for analysis.

### 2.2 Definition and variables

Sepsis was defined according to the Third International Consensus Definitions for Sepsis and Septic Shock (sepsis-3) (1, 21), with a Sequential Organ Failure Assessment (SOFA) score ≥ 2 and suspected infection during intensive care unit (ICU) stay. According to previous studies, a suspected infection is defined as a blood microbiological culture or the use of antibiotics during hospitalization. Blood microbiological culture or antibiotic use during hospitalization were considered to suggest a suspected infection. This study focused on the ratio of absolute lymphocyte levels to high-density lipoprotein (HDL) levels; thus, we excluded patients who did not have a record of these two indicators during their hospital stay. We extracted the first records of the absolute values of lymphocytes and HDL after hospitalization. This selection was made to mitigate the impact of ICU interventions, considering that they were modest short-term blood alterations. Finally, most cases of sepsis were excluded, with only 1027, 148, and 280 patients included in the MIMIC-IV, Amsterdam UMCdb, and SICdb sets, respectively. Privacy-sensitive data were de-identified in all databases, making them publicly accessible and ethically exempt.

Clinical characteristic variables had different data formats and different extraction strategies were used to extract the variables, thus maximizing their ability to reflect disease severity. Demographic data such as age, sex, and body mass index (BMI) were extracted only from the day of admission to the ICU because of their limited short-term variability. We used the codes of the International Statistical Classification of Diseases for the extraction of comorbidity data; however, owing to the limitations of the databases, we only extracted comorbidity data from the MIMIC-IV database. The comorbidities extracted from the MIMIC-IV database included congestive heart failure, diabetes, severe liver disease, kidney disease, and intrapulmonary sepsis, based on the site of infection. Comorbidity variables were transformed into binary forms for inclusion in the analysis. In addition, outcomes and treatments were extracted as binary measures. However, because some laboratory indicators and vital signs had relatively short timeliness, we extracted values that represented the current physiological state of the patient in a particular direction. The directions and detailed descriptions of all extracted variables are shown in **Table S1**. The primary clinical outcome in this study was 90-day mortality, and secondary outcomes included 7-day mortality, 28-day mortality, and length of ICU stay.

### 2.3 Statistical analysis

Numerical variables were represented as medians and interquartile ranges, and comparisons were performed using the Mann-Whitney U-test. Binary variables were described as counts and percentages, and comparisons were performed using either chi-squared or Fisher’s exact tests. **Figure S1** shows that the main missing data in the MIMIC-IV database were partial pressure of oxygen (PaO_2_) and partial pressure of carbon dioxide (PaCO_2_); however, the proportion of missing variables included was considered acceptable at 0.5. For the validation sets, the missing data conditions were relaxed, and variables with missing ratios > 0.5 were not included.

**Figure 1:**
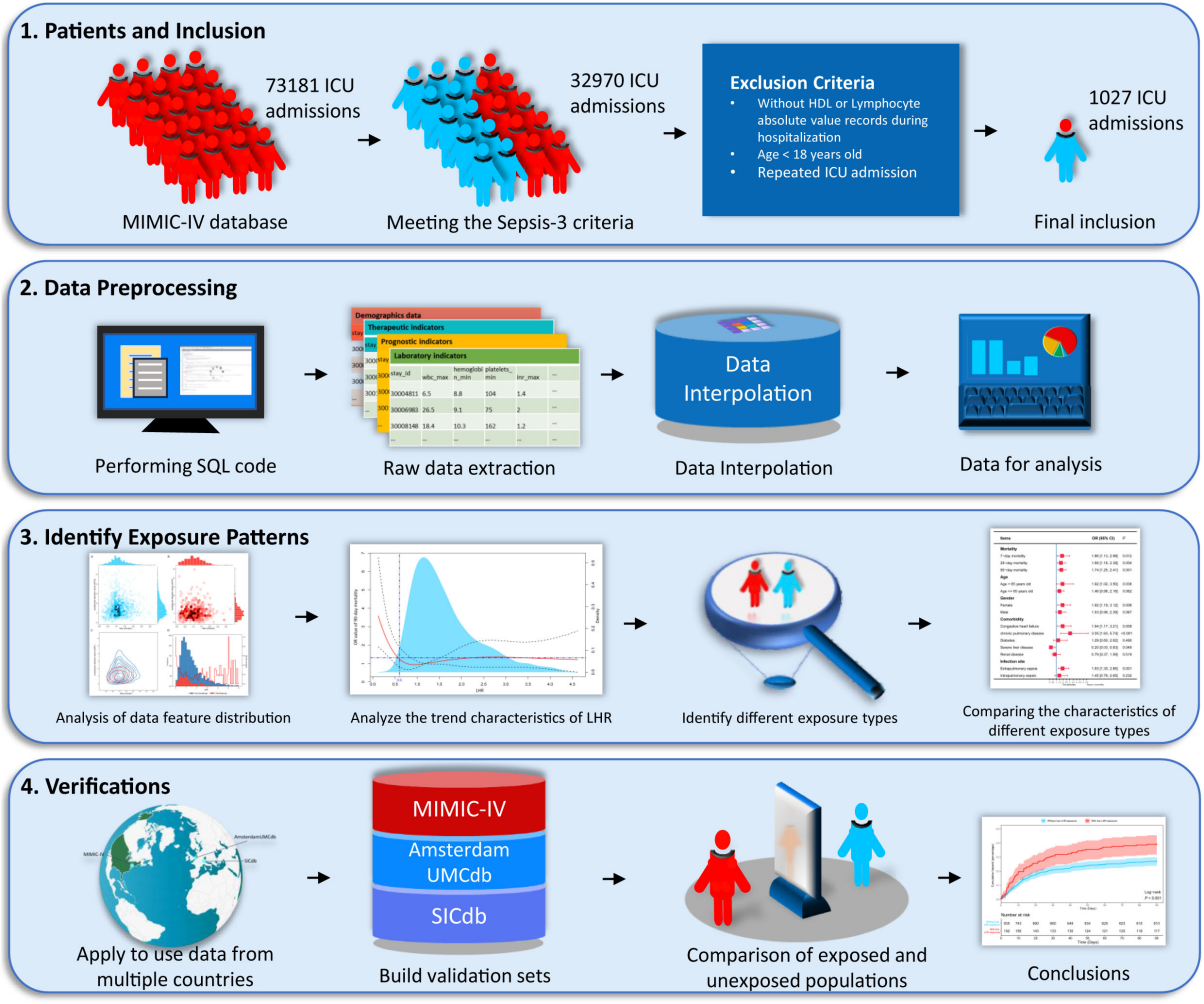
The research steps of this study.

First, we analyzed the data distribution and patterns using the MIMIC-IV database. To mitigate the effect of missing data, we used multiple imputations to interpolate the adjusted variables. We then employed logical regression to establish the relationship between various variables and 90-day mortality rates. To simplify the model, we excluded collinearity using the variance inflation factor, gradually excluded adjusted variables using a stepwise method, and measured the goodness of fit of the model using the Hosmer-Lemeshow test. We described the dose-effect relationship between LHR and the 90-day mortality rate using a restricted cubic spline plot based on multivariate logistic regression. Furthermore, we determined the dose of low LHR threshold by considering changes in odds ratio, population density, and clinical applicability. Following determination of the threshold, a validation process was conducted from various perspectives. We obtained two groups of low LHR-exposed and unexposed patients using propensity score matching (PSM) and inverse probability weighting (IPW) to ensure a balanced baseline at admission, making clinical outcomes more comparable. In addition, we compared the exposed and unexposed patients from two different datasets. Unfortunately, due to sample size limitations, PSM and IPW did not obtain sufficiently matched samples in the two datasets. We constructed a simple flowchart, shown in **Figure 1**, to describe the research process.

Standard query languages were used to construct the research physics views, and related analyses and drawings were performed using R (version 4.1.3) and Python (version 3.7.3). Statistical significance was determined if the p value was < 0.05.

## 3 Results

### 3.1 Baseline data of included patients

A total of 1027 sepsis patients with complete absolute lymphocyte values and HDL records from the MIMIC-IV database were included, as shown in **Table 1**. The median age of the population was 67 years, and 60.3% of the patients were male. Regarding short-term prognostic indicators, the median length of stay (LOS) in the ICU was 5.8 days, and the 90-day mortality rate was 29.2%. The demographics varied slightly between the two validation sets. The Amsterdam UMCdb validation set had a median population age of 65 years, with 78.4% of the sample being male and an average LOS of 0.9 days. The median age of the SICdb validation set population was 70 years, 69.3% were male, and the 90-day mortality rate was 17.9%.

**Table 1:** Baseline data of included patients in the MIMIC-IV data set.

Items	Overall	Without Low LHR group	With Low LHR group	*P* value
	1027	835	192	
Age (years old)	67 (57, 79)	66 (56, 77)	73 (60, 84)	<0.001
Male (%)	619 (60.3)	516 (61.8)	103 (53.6)	0.046
BMI (kg/m^2^)	28.3 (24.5, 32.5)	28.6 (24.9, 33.1)	26.8 (23.1, 30.7)	<0.001
Congestive heart failure (%)	370 (36.0)	287 (34.4)	83 (43.2)	0.026
Chronic pulmonary disease (%)	250 (24.3)	197 (23.6)	53 (27.6)	0.283
Diabetes (%)	329 (32.0)	289 (34.6)	40 (20.8)	<0.001
Severe liver disease (%)	74 (7.2)	60 (7.2)	14 (7.3)	1.000
Renal disease (%)	214 (20.8)	170 (20.4)	44 (22.9)	0.491
Intrapulmonary sepsis (%)	308 (30.0)	256 (30.7)	52 (27.1)	0.375
SOFA (score)	5 (3, 8)	5 (3, 8)	5 (3, 7)	0.359
SAPS II (score)	38 (31, 48)	37 (30, 48)	40 (34, 48)	0.003
WBC (×10^9^/L)	13.9 (10.1, 18.2)	14.1 (10.5, 18.4)	12.7 (8.8, 16.8)	<0.001
Hemoglobin (g/L)	10.5 (8.8, 12.2)	10.5 (8.8, 12.2)	10.6 (8.7, 11.9)	0.688
Platelet (×10^9^/L)	167 (117, 232)	169 (120, 232)	160 (105, 222)	0.131
INR	1.3 (1.1, 1.6)	1.3 (1.2, 1.6)	1.2 (1.1, 1.5)	0.002
Creatinine (mg/dL)	1.2 (0.9, 1.8)	1.2 (0.9, 1.8)	1.3 (0.9, 1.8)	0.219
PaO_2_ (mmHg)	70 (45, 98)	71 (46, 98)	62 (42, 95)	0.031
PaCO_2_ (mmHg)	45 (39, 52)	45 (39, 52)	45 (38, 53)	0.389
Base excess (mmol/L)	-2.0 (-6.0, 0.0)	-2.0 (-6.0, 0.0)	-2.0 (-5.0, 1.0)	0.637
HDL (mmol/L)	1.0 (0.7, 1.3)	0.9 (0.6, 1.2)	1.3 (1.0, 1.7)	<0.001
ABS of lymphocytes (×10^9^/L)	1.2 (0.7, 1.7)	1.3 (1.0, 1.9)	0.5 (0.3, 0.7)	<0.001
Heart rate (/bpm)	85 (75, 95)	85 (75, 95)	82 (74, 94)	0.142
Respiratory rate (/bpm)	19 (17, 22)	19 (17, 22)	19 (17, 23)	0.107
SBP (mmHg)	118 (107, 132)	117 (107, 131)	123 (108, 136)	0.013
DBP (mmHg)	63 (56, 71)	63 (56, 70)	64 (55, 75)	0.092
Body temperature (°C)	36.9 (36.6, 37.3)	36.9 (36.6, 37.3)	36.8 (36.6, 37.2)	0.226
Urine output (mL)	1550 (932, 2348)	1570 (956, 2378)	1402 (782, 2120)	0.031
Vasopressor (%)	407 (39.6)	354 (42.4)	53 (27.6)	<0.001
Ventilation (%)	600 (58.4)	502 (60.1)	98 (51.0)	0.026
Length of ICU stay (day)	5.8 (2.7, 12.2)	6.0 (2.6, 12.1)	5.3 (2.9, 12.2)	0.913
7-day mortality (%)	91 (8.9)	65 (7.8)	26 (13.5)	0.017
28-day mortality (%)	229 (22.3)	171 (20.5)	58 (30.2)	0.005
90-day mortality (%)	300 (29.2)	225 (26.9)	75 (39.1)	0.001

LHR, absolute value of lymphocytes to high-density lipoprotein ratio; BMI, body mass index; SOFA, Sequential Organ Failure Score; SAPS II, Simplified Acute Physiology Score II; WBC, white blood cell count; INR, international standardized ratio; PaO2, partial pressure of oxygen; PaCO2, partial pressure of carbon dioxide; HDL, high-density lipoprotein; ABS, absolute value; SBP, systolic pressure; DBP, diastolic pressure; Vasopressor, use of vasopressors on first day admitted to ICU; Ventilation, use of ventilation on first day admitted to ICU; ICU, Intensive Care Unit.

### 3.2 The relationship between the distribution of LHR and 90-day mortality

**Figure 2:**
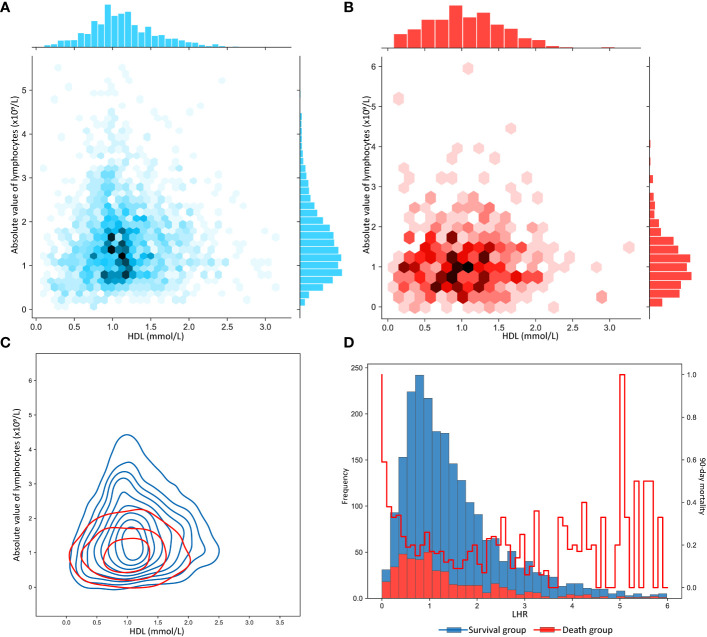
**(A)** Distribution characteristics of the absolute value of lymphocytes and of high-density lipoprotein in patients with sepsis who survived more than 90 days. **(B)** Distribution characteristics of the absolute value of lymphocytes and of high-density lipoprotein in patients with sepsis who survived less than 90 days. **(C)** Contour plot of absolute value of lymphocytes and high-density lipoprotein in sepsis patients with 90-day survival and death. **(D)** The bar chart plots the frequency distribution characteristics of LHR in sepsis patients who survived and died within 90 days, where the line graph shows the distribution of the 90-day mortality rate. LHR, absolute value of lymphocytes to high-density lipoprotein ratio.

**Figure 3:**
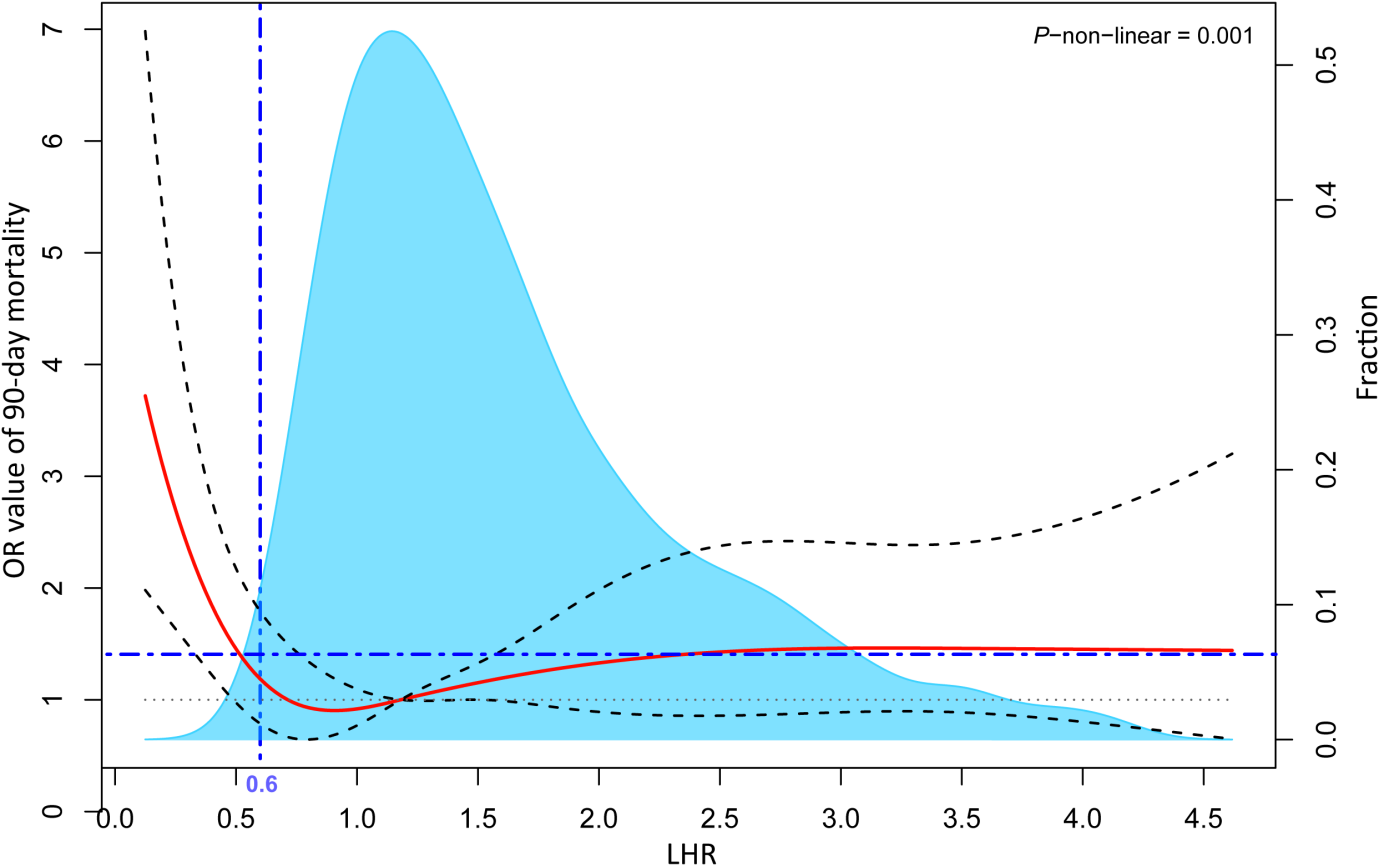
Multivariable adjusted odds ratios for the 90-day mortality rates based on the continuous LHR levels. The solid red lines represent multivariable adjusted odds ratios while the dashed grey lines depict the 95% confidence intervals derived from restricted cubic spline regressions. The blue area shows the proportion of the population with varying levels of LHR. The analysis is based on multivariable logistic regression. Its adjusted variables are screened by variance inflation factor (< 2) and stepwise method (*P* < 0.1), including age, diabetes, intrapulmonary sepsis, WBC, INR, respiratory rate, SBP, urine output, and its goodness of fit is tested by Hosmer-Lemeshow test (*P* > 0.05). LHR, absolute value of lymphocytes to high-density lipoprotein ratio; WBC, white blood cell count; INR, international standardized ratio; SBP, systolic pressure.

As shown in **Table S3**, LHR distributions of survival and death groups were compared in three datasets. Interestingly, although the LHR differences between the survival and death group were not significant in the three datasets, they were moderately higher than those in the death group. To further describe the difference, as shown in **Figures 2A, B**, we visualized the interactive distribution between the absolute values of lymphocytes and HDL. As shown, the interactive distribution of absolute values of lymphocytes and HDL is more dispersed in the death group, while the survival group is more concentrated. The absolute lymphocyte count was approximately 1 ×10^9^/L in the survivors and below this level in the deceased. The contour map, which shows the absolute values of lymphocytes and HDL, clearly exhibits a considerable shift in the density of the distributions (**Figure 2C**). These results indicate a potential correlation between the two patterns of human biological indicators and 90-day mortality of patients with sepsis. We then represented the distribution of surviving patients and deaths within 90 days using a bar chart, and the variation in the 90-day mortality of LHR at different distribution intervals using a line chart (**Figure 2D**). We compared the distribution of patients who survived and those who died over the 90-day period using bar graphs. Moreover, we represent the mortality rate changes in the LHR under different distribution intervals using line graphs. The results indicated a significant increase in the mortality rate when the LHR was either too low or too high. However, when the LHR was too high, there were significant fluctuations in the mortality rates. Multivariate adjusted logistic regression was used to construct a restricted cubic spline graph to accurately visualize the relationship between LHR and mortality. **Figure 3** shows a significant non-linear relationship between the LHR and mortality in sepsis patients (*P* = 0.001). Considering the impact of high LHR, population distribution density, and practicality of the clinical application, we finally defined patients with LHR values of ≤ 0.6 as low LHR threshold.

### 3.3 Sensitivity analysis and subgroup analysis of low LHR threshold

**Figure 4:**
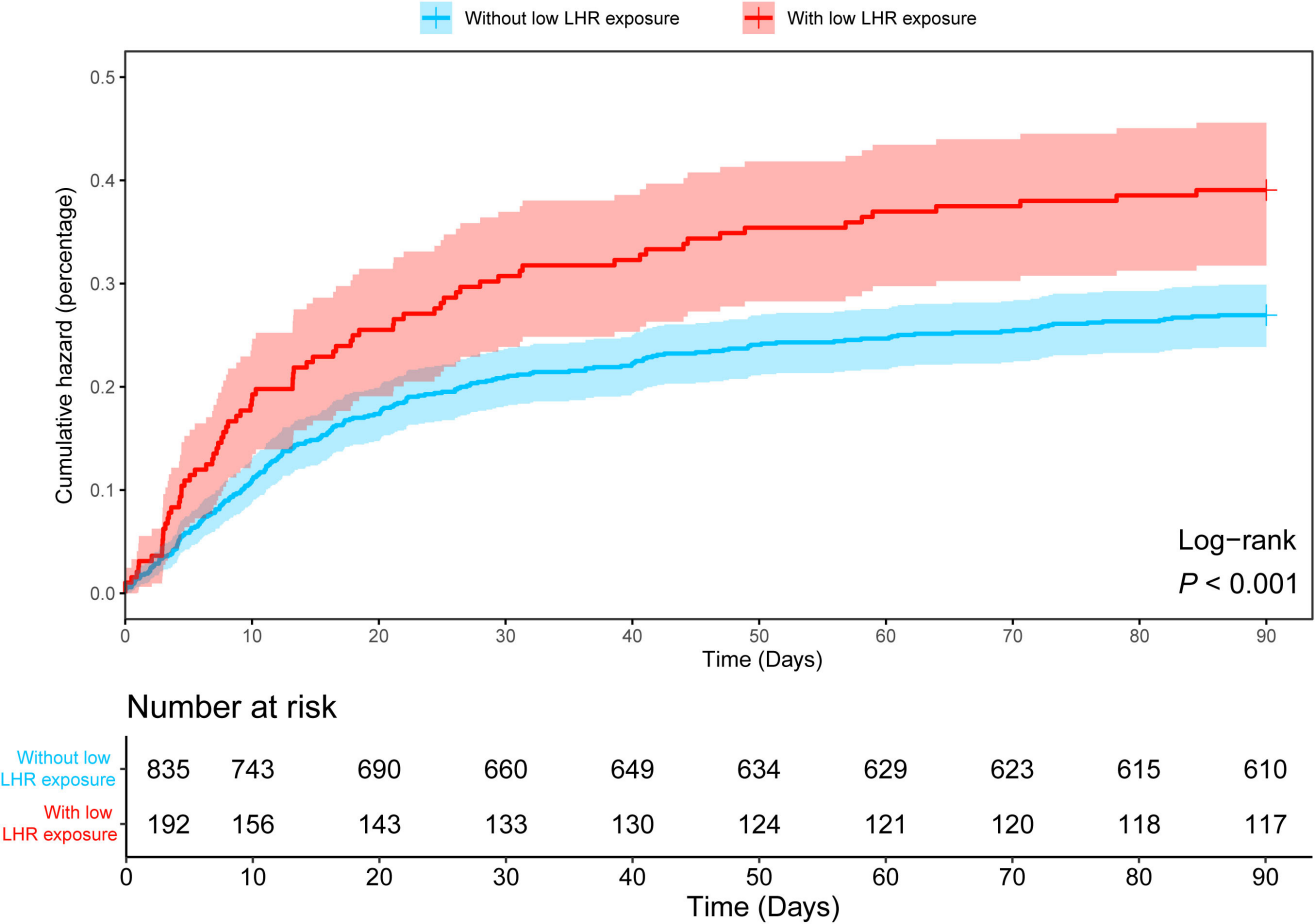
Kaplan-Meier curve showing differences in 90-day cumulative mortality in sepsis patients with and without low LHR group. LHR, absolute value of lymphocytes to high-density lipoprotein ratio.

As shown in **Table 1**, in the MIMIC-IV database, patients with low LHR were older but physically thinner (*P* < 0.001), which was consistent in the two validation sets (**Tables S3**, **S4**). Moreover, an increase in 90-day mortality was observed in sepsis patients with low LHR threshold (**Figure 4**). However, differences in short-term mortality, such as 7-day mortality, were not stable across the validation sets, whereas mortality gradually increased in the lower LHR group over time (**Tables 1**, **S3**, **S4**; **Figure 4**). In general, we observed that low LHR group was associated with poor clinical outcomes. This was observed not only in mortality but also in several indicators that may reflect the seriousness of the illness. Our results consistently showed that low LHR group was associated with lower WBC and platelet counts and higher SAPS scores across all validation sets (**Tables 1**, **S3**, **S4**). Interesting odds ratio (OR) characteristics were noted in the subgroup analysis. Among patients aged < 65 years, female patients, and patients with chronic lung disease, the OR for 90-day mortality was significantly higher (**Figure S2**, *P* < 0.05).

### 3.4 Methodology validation and external validation

The grouping of the enrolled patients may have been biased owing to unbalanced baseline data. Thus, we used PSM and IPW to reduce baseline imbalance. As shown in **Figure S3**, both PSM and IPW significantly reduced the imbalance of unmatched data and increased comparability. After PSM matching, the OR for 90-day mortality was 1.56, with a p-value of < 0.05. In the Amsterdam UMCdb and SICdb validation sets, the OR values for 90-day mortality were 7.00 and 2.26, respectively, indicating that a low LHR group has a warning value in patients with sepsis from different geographies (**Figure S4**).

## 4 Discussion

The main conclusions drawn from this multicenter retrospective study are as follows:(i) Patients who died within 90 days of sepsis had lower lymphocyte counts and LHR than those who survived. (ii) Septic patients exposed to a low LHR (LHR ≤ 0.6) had an increased chance of septicemia 90-day mortality. (iii) The conclusion of increased 90-day mortality in patients with sepsis with low LHR remains true after validation using different datasets or matching methods. To the best of our knowledge, this is the first clinical study to focus on the relationship between LHR, a new inflammatory marker, and mortality in sepsis.

Patients who die of sepsis have significant post-invasive immunosuppression, as evidenced by lymphopenia (22, 23). Flow cytometric phenotyping of circulating leukocyte subpopulations in patients with sepsis have revealed that the adaptive immune response to sepsis produces immunosuppression involving almost all lymphocyte subpopulations (24). The possible mechanisms involved are related to reduced expression of T-bet, GATA binding protein 3 (GATA 3) and retinoic acid receptor-associated orphan receptor-γt (RORγt) (24, 25), stabilization of the expression of the TReg-cell-associated transcription factor forkhead box P3 (FOXP 3) (26), and increased PD 1 expression on T cells (27). The degree of reduction in lymphocyte count reflects the degree of immunosuppression in sepsis (28). A prospective study that included 92 patients admitted to the intensive care unit for severe sepsis or septic shock found that a persistent decrease in lymphocyte count was associated with increased mortality in patients with sepsis (6). Another study of 753 patients extracted from a French ICU database found that a decrease in the absolute lymphocyte count at baseline was associated with an increased incidence of ICU-acquired infections and 28-day mortality (28). In this study, we found that patients in the MIMIC-IV database who died within 90 days of developing sepsis had lower lymphocyte counts than those who survived. HDL is a vasoprotective factor that plays a protective role against the development of atherosclerosis (29). However, HDL is also involved in the pathological processes of inflammation and acute inflammatory diseases, and plays multiple protective roles in the pathophysiology of sepsis. HDL acts as an LPS neutralizer and binds to it to form HDL-LPS which attenuates LPS-TLR 4 inflammatory signalling in macrophages and acts in conjunction with its scavenger receptor BI (SR-BI) to promote LPS clearance (30). In addition to the inactivation of bacterial lipopolysaccharides and other biohazards, HDL prevents oxidative stress and inflammation, and inhibits thrombosis by promoting cholesterol efflux from cells and upregulating the transcriptional regulator ATF3 (31, 32). The HDL levels of the included study participants were at or below the lower limit of normal, reflecting the presence of potential sepsis susceptibility factors.

LHR is a new inflammatory marker and current studies suggest that it may be an effective inflammatory marker for predicting the presence and severity of MetS in China (17). However, our findings reveal for the first time that LHR ≤ 0.6 is a potentially important biomarker of increased risk of death from sepsis. Our statistical analysis of the MIMIC-IV database concluded that exposure to a low LHR is a risk factor for an increased risk of death from sepsis. To further increase the credibility of this conclusion, we performed PSM and IPW matching on the MIMIC-IV data and further validated it using the Amsterdam UMCdb and SICdb datasets. The results obtained were consistent, which shows that our findings are credible and somewhat generalizable. We found that patients in the low LHR-exposed group tended to be older and thinner (P < 0.001), suggesting an underlying increase in comorbidities and relatively poor nutritional status, which are risk factors for increased sepsis mortality (33). The low LHR-exposed group also had a combination of lower white blood cell and platelet counts, and higher SAPS scores, suggesting a higher cumulative risk of fatality, lower survival, and more risk factors (34, 35). In addition, the mortality rate in the low LHR-exposed group increased over time, which may be related to the increased incidence of hospital-acquired infections due to prolonged hospitalization and reactivation of latent viruses (cytomegalovirus and herpes simplex virus) (36). In addition, we found that the predictive value of low LHR group was more pronounced in female sepsis patients aged < 65 years with comorbid chronic lung disease by subgroup analyses, which demonstrated significantly higher odds ratios for 90-day mortality. A possible explanation for this may be that women aged < 65 years generally have higher levels of estrogen, which possesses proinflammatory properties that can exacerbate immune damage (37). More importantly, previous studies have demonstrated that women with chronic lung disease have faster disease progression and higher mortality rates (38).

Lymphocyte count and HDL are inexpensive, common, and readily available laboratory markers in clinical practice. Therefore, low LHR group as a risk factor for increased mortality in sepsis has implications for early intervention in sepsis and the reduction of hospitalization costs. However, there are several limitations in interpreting the results of our study. First, although we used several algorithms to interpolate the study data, our data were still deficient, resulting in missing values, which may have led to a small amount of bias in the data analysis and modelling results. Second, in terms of the target population, we used databases from multiple national research centers, but this only included white people in developed countries; populations in developing countries, or different ethnicities, were thoughtless, and potential influences such as the living environment, lifestyles, and economic bases were not taken into account. Randomized controlled clinical trials or further validations using larger samples may help increase the reliability of the conclusions.

In conclusion, high LHR tends to increase mortality in patients with sepsis, however additional data on LHR patterns are necessary, and further research is required.

## Data availability statement

The original contributions presented in the study are included in the article/**Supplementary Material**. Further inquiries can be directed to the corresponding author.

## Author contributions

WL: Conceptualization, Data curation, Formal Analysis, Methodology, Resources, Validation, Visualization, Writing – original draft. QT: Conceptualization, Formal Analysis, Writing – original draft, Writing – review & editing, Investigation. JX: Formal Analysis, Investigation, Writing – original draft. YD: Investigation, Writing – review & editing. TP: Investigation, Writing – review & editing. YW: Investigation, Writing – review & editing. XZ: Conceptualization, Funding acquisition, Resources, Supervision, Writing – review & editing.

## Supplementary material

Supplementary Figure 1 The distribution of missing Values in the MIMIC-IV, Amsterdam UMCdb and SICdb sets. BMI, body mass index; PaO2, arterial oxygen partial pressure; PaCO2, arterial carbon dioxide partial pressure; SBP, systolic pressure; DBP, diastolic pressure.

Supplementary Figure 2 The odds ratio in sepsis patients with low LHR exposure in different subgroups in the MIMIC-IV set. Subgroup OR values were determined by comparison with 90-day mortality using logistic regression, except for the first two items. LHR, absolute value of lymphocytes to high-density lipoprotein ratio; OR, odds ratio.

Supplementary Figure 3 The line plots showing the standardized mean difference (SMD) of unmatched patients, patients matched using propensity score matching (PSM), and patients matched using inverse probability weighting (IPW) based on baseline data. The plot represents data for patients included in the MIMIC-IV data set. BMI, body mass index; SOFA, Sequential Organ Failure Score; SAPS II, Simplified Acute Physiology Score II; WBC, white blood cell count; INR, international standardized ratio; PaO2, arterial oxygen partial pressure; PaCO2, arterial carbon dioxide partial pressure; SBP, systolic pressure; DBP, diastolic pressure; Vasopressor, use of vasopressors on first day admitted to ICU; Ventilation, use of ventilation on first day admitted to ICU; ICU, Intensive Care Unit.

Supplementary Figure 4 Forest plots comparing the 90-day mortality rates of sepsis patients across various data sets or matched with different methods. PSM, propensity score matching;IPW, inverse probability weighting.
