# Supplementary figures and images for: Low lymphocyte to high-density lipoprotein ratio predicts mortality in sepsis patients
Source: Front Immunol. 2023 Oct 12;14:1279291. doi: 10.3389/fimmu.2023.1279291 (PMC10601636; doi:10.3389/fimmu.2023.1279291)

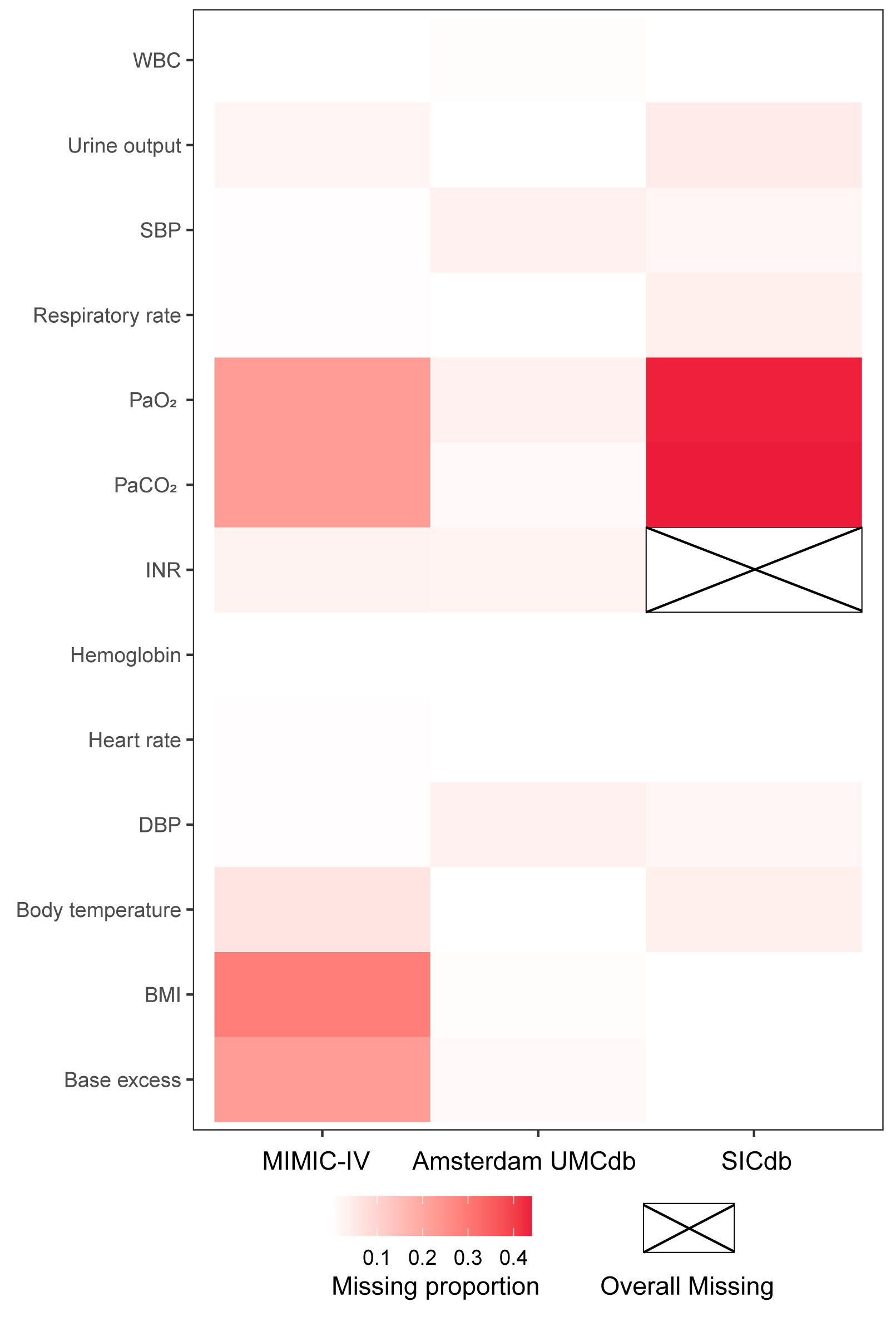

Supplement: Supplementary Figure 1 — The distribution of missing Values in the MIMIC-IV, Amsterdam UMCdb and SICdb sets. BMI, body mass index; PaO2, arterial oxygen partial pressure; PaCO2, arterial carbon dioxide partial pressure; SBP, systolic pressure; DBP, diastolic pressure. [file Image_1.tif]

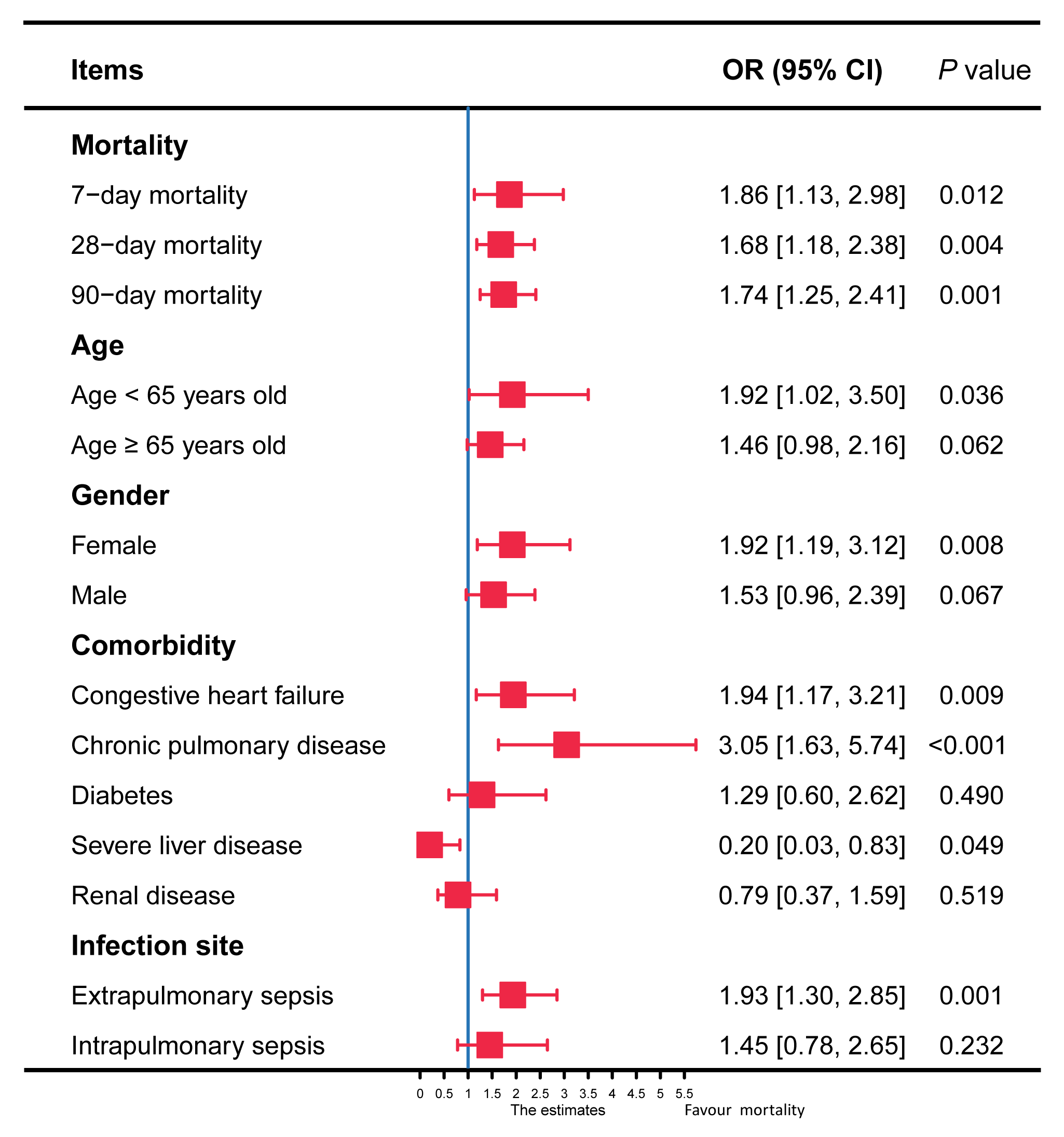

Supplement: Supplementary Figure 2 — The odds ratio in sepsis patients with low LHR exposure in different subgroups in the MIMIC-IV set. Subgroup OR values were determined by comparison with 90-day mortality using logistic regression, except for the first two items. LHR, absolute value of lymphocytes to high-density lipoprotein ratio; OR, odds ratio. [file Image_2.tif]

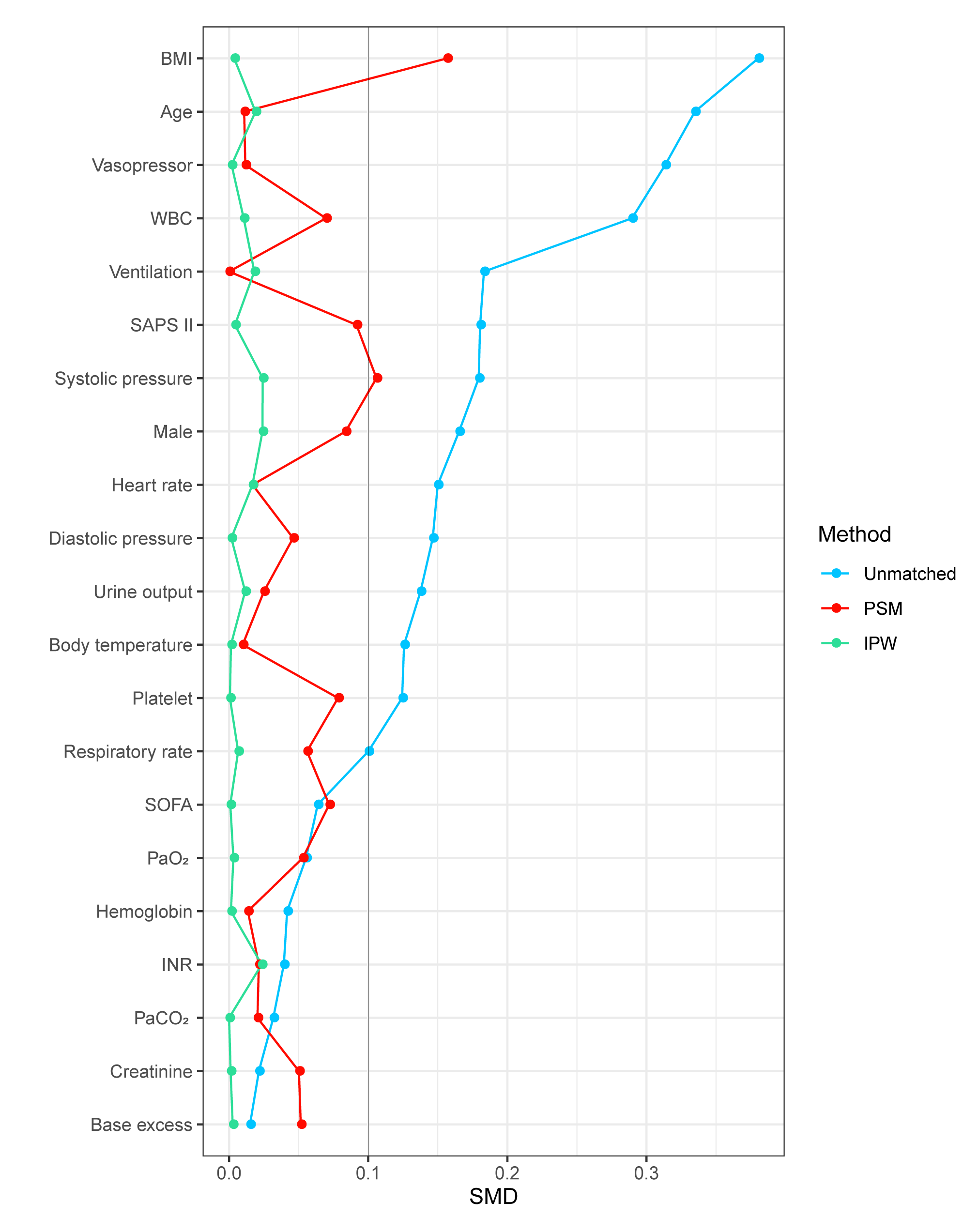

Supplement: Supplementary Figure 3 — The line plots showing the standardized mean difference (SMD) of unmatched patients, patients matched using propensity score matching (PSM), and patients matched using inverse probability weighting (IPW) based on baseline data. The plot represents data for patients included in the MIMIC-IV data set. BMI, body mass index; SOFA, Sequential Organ Failure Score; SAPS II, Simplified Acute Physiology Score II; WBC, white blood cell count; INR, international standardized ratio; PaO2, arterial oxygen partial pressure; PaCO2, arterial carbon dioxide partial pressure; SBP, systolic pressure; DBP, diastolic pressure; Vasopressor, use of vasopressors on first day admitted to ICU; Ventilation, use of ventilation on first day admitted to ICU; ICU, Intensive Care Unit. [file Image_3.tif]

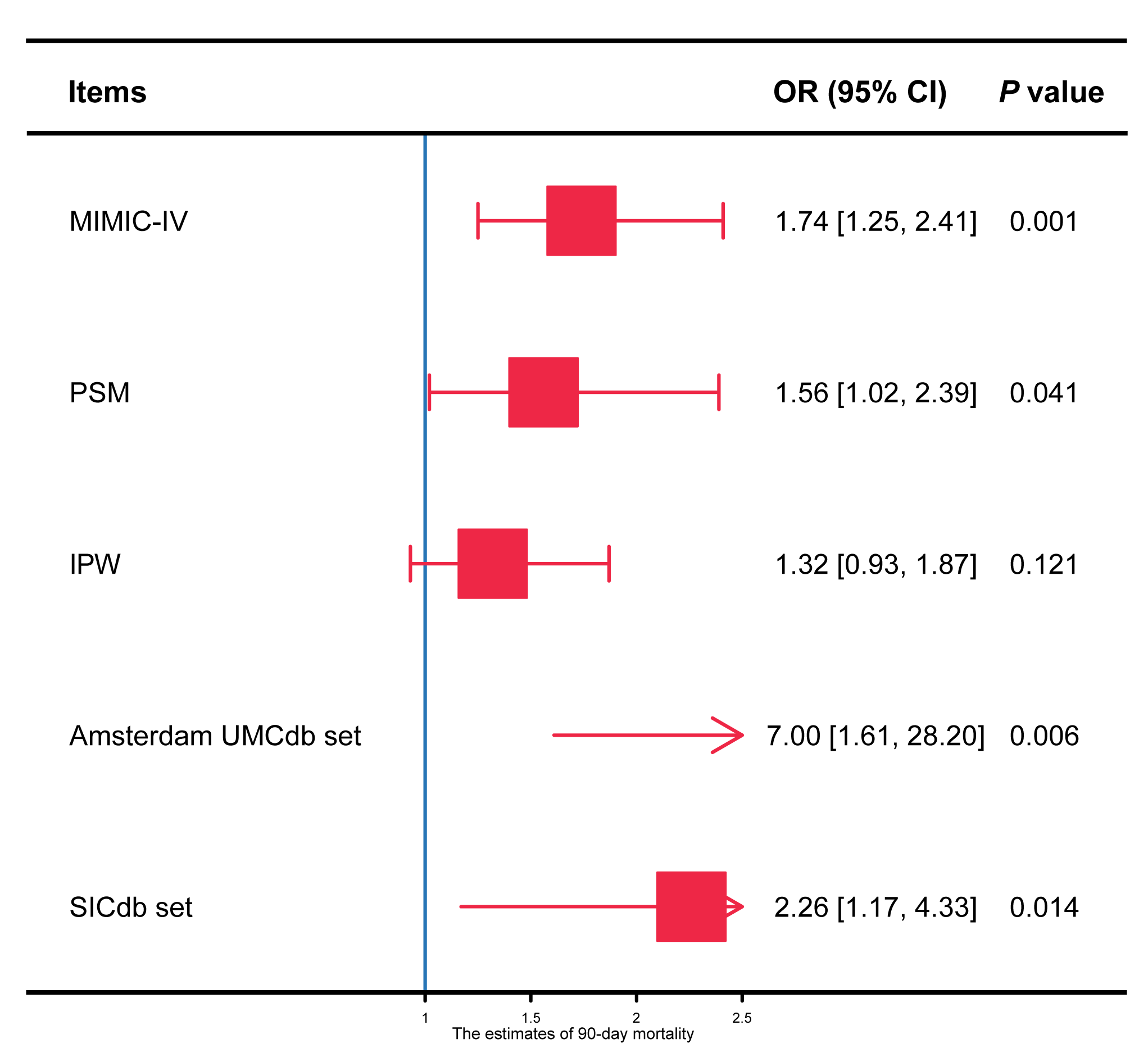

Supplement: Supplementary Figure 4 — Forest plots comparing the 90-day mortality rates of sepsis patients across various data sets or matched with different methods. PSM, propensity score matching;IPW, inverse probability weighting. [file Image_4.tif]
