# Supplementary material for: Low lymphocyte to high-density lipoprotein ratio predicts mortality in sepsis patients
Source: Front Immunol. 2023 Oct 12;14:1279291. doi: 10.3389/fimmu.2023.1279291 (PMC10601636; doi:10.3389/fimmu.2023.1279291)
Supplement: Supplementary file 5 [file Table_1.docx]

Table S1. All relevant variable data types, directions and abbreviations.

| Items | Data types | Extraction directions | Abbreviations |
| --- | --- | --- | --- |
| Age | Numerical | - | Age |
| Male | Binary | - | Male |
| Body mass index | Numerical | - | BMI |
| Congestive heart failure | Binary | - | - |
| Chronic pulmonary disease | Binary | - | - |
| Diabetes | Binary | - | - |
| Severe liver disease | Binary | - | - |
| Renal disease | Binary | - | - |
| Intrapulmonary sepsis | Binary | - | - |
| Sequential Organ Failure Score | Numerical | Max on the first day | SOFA |
| Simplified Acute Physiology Score II | Numerical | Max on the first day | SAPS III |
| Simplified Acute Physiology Score III | Numerical | Max on the first day | SAPS III |
| White blood cell count | Numerical | Max on the first day | WBC |
| Hemoglobin | Numerical | Min on the first day | Hemoglobin |
| Platelet | Numerical | Min on the first day | Platelets |
| International standardized ratio | Numerical | Max on the first day | INR |
| Creatinine | Numerical | Max on the first day | Creatinine |
| Partial pressure of oxygen | Numerical | Min on the first day | PaO_2_ |
| Partial pressure of carbon dioxide | Numerical | Max on the first day | PaCO_2_ |
| Base excess | Numerical | Min on the first day | - |
| High density lipoprotein | Numerical | First record | HDL |
| Absolute value of lymphocytes | Numerical | First record | ABS of lymphocytes |
| Heart rate | Numerical | Mean on the first day | - |
| Respiratory rate | Numerical | Mean on the first day | - |
| Systolic pressure | Numerical | Mean on the first day | SBP |
| Diastolic pressure | Numerical | Mean on the first day | DBP |
| Body temperature | Numerical | Mean on the first day | - |
| Urine output | Numerical | Cumulative volume on the first day | - |
| Use of vasopressors on first day admitted to ICU | Binary | - | Vasopressor |
| Use of ventilation on first day admitted to ICU | Binary | - | Ventilation |
| Length of ICU stay | Numerical | - | LOS |
| 7-day mortality (%) | Binary | - | - |
| 28-day mortality (%) | Binary | - | - |
| 90-day mortality (%) | Binary | - | - |

Table S2. Distribution and Comparison of LHR in the Death and Survival Groups.

| Data set | Survival group | Death group^a^ | *P* value |
| --- | --- | --- | --- |
|  | 138 | 10 |  |
| MIMIC-IV | 1.3 (0.8, 2.1) | 1.1 (0.6, 2.2) | 0.097 |
| Amsterdam UMCdb | 1.8 (1.3, 2.4) | 1.1 (0.6, 2.2) | 0.050 |
| SICdb | 1.0 (0.7, 1.7) | 0.8 (0.5, 1.6) | 0.211 |

LHR, absolute value of lymphocytes to high-density lipoprotein ratio.

a: The death group represents patients who died within 90 days.

Table S3. Baseline data of included patients in the Amsterdam UMCdb data set.

| Items | Overall | Without Low LHR group | With Low LHR group | *P* value |
| --- | --- | --- | --- | --- |
|  | 148 | 138 | 10 |  |
| Age (years old) | 65 (55, 75) | 65 (55, 75) | 65 (58, 75) | 0.794 |
| Male (%) | 116 (78.4) | 107 (77.5) | 9 (90.0) | 0.598 |
| BMI (kg/m^2^) | 27.8 (24.5, 31.0) | 27.8 (24.5, 31.0) | 24.8 (21.9, 27.8) | 0.145 |
| SOFA (score) | 6 (5, 8) | 6 (5, 8) | 5 (4, 11) | 0.896 |
| SAPS II (score) | 33 (27, 40) | 33 (27, 39) | 38.0 (30, 47) | 0.167 |
| WBC (×10^9^/L) | 16.8 (12.9, 20.9) | 17.1 (13.6, 21.0) | 11.7 (5.5, 14.3) | 0.004 |
| Hemoglobin (g/L) | 6.1 (5.3, 7.1) | 6.2 (5.3, 7.2) | 5.2 (4.3, 6.4) | 0.090 |
| Platelet (×10^9^/L) | 152 (107, 194) | 152 (109, 196) | 152 (92, 173) | 0.477 |
| INR | 1.5 (1.4, 1.7) | 1.5 (1.4, 1.7) | 1.4 (1.3, 1.5) | 0.446 |
| Creatinine (mg/dL) | 89 (73, 120) | 89 (73, 112) | 147 (92, 282) | 0.073 |
| PaO_2_ (mmHg) | 40 (35, 72) | 40 (34, 72) | 58 (42, 68) | 0.300 |
| PaCO_2_ (mmHg) | 46 (43, 51) | 46 (44, 51) | 43 (33, 51) | 0.250 |
| Base excess (mmol/L) | 0.4 (0.1, 1.8) | 0.4 (0.1, 1.7) | 1.1 (0.3, 4.4) | 0.153 |
| HDL (mmol/L) | 1.0 (0.8, 1.3) | 1.0 (0.8, 1.2) | 1.2 (1.1, 1.5) | 0.114 |
| ABS of lymphocytes (×10^9^/L) | 1.6 (1.3, 2.3) | 1.7 (1.4, 2.3) | 0.6 (0.2, 0.6) | <0.001 |
| Heart rate (/bpm) | 81 (74, 91) | 81 (74, 89) | 92.0 (85, 111) | 0.043 |
| Respiratory rate (/bpm) | 16 (15, 19) | 16 (14, 18) | 23 (18, 24) | 0.006 |
| SBP (mmol/L) | 117 (107, 130) | 117 (107, 130) | 116 (115, 127) | 0.612 |
| DBP (mmol/L) | 59 (55, 66) | 59 (56, 66) | 52 (51, 65) | 0.365 |
| Body temperature (℃) | 36.6 (36.1, 36.8) | 36.5 (36.1, 36.8) | 36.9 (36.3, 37.3) | 0.120 |
| Urine output (mL) | 1780 (1298, 2573) | 1793 (1312, 2581) | 1445 (1128, 2166) | 0.313 |
| Vasopressor (%) | 112 (75.7) | 106 (76.8) | 6 (60.0) | 0.415 |
| Ventilation (%) | 129 (87.2) | 124 (89.9) | 5 (50.0) | 0.002 |
| Length of ICU stay (day) | 0.9 (0.9, 1.6) | 0.9 (0.8, 1.1) | 9.2 (1.2, 14.2) | 0.003 |
| 7-day mortality (%) | 9 (6.1) | 7 (5.1) | 2 (20.0) | 0.222 |
| 28-day mortality (%) | 13 (8.8) | 9 (6.5) | 4 (40.0) | 0.002 |
| 90-day mortality (%) | 16 (10.8) | 12 (8.7) | 4 (40.0) | 0.011 |

LHR, absolute value of lymphocytes to high-density lipoprotein ratio; BMI, body mass index; SOFA, Sequential Organ Failure Score; SAPS II, Simplified Acute Physiology Score II; WBC, white blood cell count; INR, international standardized ratio; PaO_2,_ partial pressure of oxygen; PaCO_2,_ partial pressure of carbon dioxide; HDL, high-density lipoprotein; ABS, absolute value; SBP, systolic pressure; DBP, diastolic pressure; Vasopressor, use of vasopressors on first day admitted to ICU; Ventilation, use of ventilation on first day admitted to ICU; ICU, Intensive Care Unit.

Table S4. Baseline data of included patients in the SICdb data set.

| Items | Overall | Without Low LHR group | With Low LHR group | *P* value |
| --- | --- | --- | --- | --- |
|  | 280 | 212 | 68 |  |
| Age (years old) | 70 (60, 80) | 70 (60, 80) | 75 (69, 80) | 0.017 |
| Male (%) | 194 (69.3) | 154 (72.6) | 40 (58.8) | 0.046 |
| BMI (kg/m^2^) | 26.3 (23.9, 29.4) | 27.3 (24.2, 29.4) | 24.7 (20.2, 27.7) | 0.001 |
| SOFA^a^ (score) | 3 (2, 4) | 3 (2, 4) | 3 (2, 5) | 0.441 |
| SAPS III^b^ (score) | 49 (42, 63) | 48 (41, 62) | 60 (47, 66) | 0.002 |
| WBC (×10^9^/L) | 14.8 (10.8, 20.9) | 14.9 (11.2, 21.7) | 13.1 (9.7, 18.0) | 0.014 |
| Hemoglobin (g/L) | 7.6 (6.8, 9.0) | 7.6 (6.8, 9.0) | 7.7 (6.9, 8.8) | 0.786 |
| Platelet (×10^9^/L) | 127 (86, 157) | 132 (90, 167) | 109 (75, 135) | 0.001 |
| Creatinine (mg/dL) | 1.5 (1.0, 2.6) | 1.5 (1.1, 2.4) | 1.6 (1.0, 2.8) | 0.681 |
| PaO_2_ (mmHg) | 68 (58, 82) | 69 (57, 81) | 66 (58, 83) | 0.898 |
| PaCO_2_ (mmHg) | 44 (39, 52) | 44 (39, 51) | 44 (39, 56) | 0.965 |
| Base excess (mmol/L) | -5.4 (-8.3, -3.2) | -5.4 (-8.3, -3.2) | -5.6 (-7.9, -3.0) | 0.612 |
| HDL (mmol/L) | 1.1 (0.8, 1.4) | 1.0 (0.7, 1.3) | 1.4 (1.1, 1.9) | <0.001 |
| ABS of lymphocytes (×10^9^/L) | 1.1 (0.8, 1.6) | 1.3 (1.0, 1.8) | 0.6 (0.5, 0.8) | <0.001 |
| Heart rate (/bpm) | 81 (75, 90) | 81 (75, 90) | 82 (73, 89) | 0.602 |
| Respiratory rate (/bpm) | 16 (14, 19) | 16 (15, 19) | 16 (14, 18) | 0.780 |
| SBP (mmol/L) | 110 (100, 122) | 110 (100, 120) | 112 (103, 127) | 0.189 |
| DBP (mmol/L) | 55 (50, 60) | 55 (51, 60) | 53 (49, 58) | 0.124 |
| Body temperature (℃) | 36.9 (36.3, 37.3) | 36.9 (36.4, 37.3) | 36.9 (36.1, 37.3) | 0.659 |
| Urine output (mL) | 1410 (760, 2018) | 1425 (820, 2030) | 1363 (684, 1899) | 0.434 |
| Vasopressor (%) | 194 (69.3) | 150 (70.8) | 44 (64.7) | 0.430 |
| Ventilation (%) | 26 (9.3) | 23 (10.8) | 3 (4.4) | 0.177 |
| Length of ICU stay (day) | 3.7 (1.9, 7.2) | 3.6 (1.8, 7.2) | 3.9 (2.0, 7.4) | 0.495 |
| 7-day mortality (%) | 10 (3.6) | 7 (3.3) | 3 (4.4) | 0.957 |
| 28-day mortality (%) | 28 (10.0) | 18 (8.5) | 10 (14.7) | 0.210 |
| 90-day mortality (%) | 50 (17.9) | 31 (14.6) | 19 (27.9) | 0.021 |

LHR, absolute value of lymphocytes to high-density lipoprotein ratio; BMI, body mass index; SOFA, Sequential Organ Failure Score; SAPS III, Simplified Acute Physiology Score III; WBC, white blood cell count; INR, international standardized ratio; PaO_2,_ partial pressure of oxygen; PaCO_2,_ partial pressure of carbon dioxide; HDL, high-density lipoprotein; ABS, absolute value; SBP, systolic pressure; DBP, diastolic pressure; Vasopressor, use of vasopressors on first day admitted to ICU; Ventilation, use of ventilation on first day admitted to ICU; ICU, Intensive Care Unit.

a, The Glasgow Coma Scale is not included in the SICdb. Therefore, the contribution of the nervous system is missing from this SOFA score. b, The Simplified Acute Physiology Score II is not included in the SICdb. For this reason, we use the original SAPS III score in the database to replace it.
